# Correlations between free fatty acids and volatile compounds in four pork cuts from Shanxia long black pigs: implications for cut-specific flavor generation

**DOI:** 10.3389/fnut.2026.1840577

**Published:** 2026-08-13

**Authors:** Li Zhang, Wen Luo, Zhen Zhang, Longyun Li, Yizhong Huang

**Affiliations:** 1 College of Life Science, Nanchang Normal University, Nanchang, China; 2 College of Forestry, Jiangxi Agricultural University, Nanchang, China; 3 School of Basic Medical Sciences, Guilin Medical University, Guilin, China

**Keywords:** Pork cuts, free fatty acids, volatile organic compounds, flavor, C20:1n9c

## Abstract

**Introduction:**

Muscle properties and meat quality vary across pork cuts, while the free fatty acids (FFAs) and volatile organic compounds (VOCs) differences among pork cuts, as well as the interrelationships between them, remain poorly understood.

**Methods:**

Four typical pork cuts were sampled. FFAs and VOCs were extracted and analyzed using gas chromatography–mass spectrometry (GC–MS). Compound identification was based on mass spectral libraries and retention indices. Odor activity values (OAVs) were calculated as the ratio of concentration to odor threshold for key volatiles. Correlation analyses were performed to explore associations between individual FFAs and differentially abundant VOCs.

**Results and discussion:**

A total of 35 FFAs and 906 VOCs were identified across the four cuts. The longissimus thoracis (LT) muscle showed a significantly higher proportion of saturated and monounsaturated fatty acids (SFAs and MUFAs) but a lower polyunsaturated fatty acid (PUFA) proportion (*p* < 0.05). According to odor activity values (OAVs), seven odor‑active compounds differed significantly among cuts, with notably higher contents in the semimembranosus (SMM) muscle. Correlation analysis revealed that several FFAs were closely associated with key VOCs, among which C20:1n9c (cis‑11‑eicosenoic acid) stood out as strongly correlated with multiple characteristic pork aroma volatiles, suggesting its critical role in shaping the overall volatile profile. These findings highlight that FFA composition not only varies by cuts but also differentially contributes to flavor formation, providing a biochemical basis for cut‑specific processing strategies and quality improvement. The results also offer a reference for future studies on flavor modulation and consumer preference alignment.

## 1 Introduction

Pork ranks among the most widely consumed meats globally (1). The Organisation for Economic Co-operation and Development (OECD) reports that China is a leading global pork consumer, with per capita intake surpassing 30 kg annually (2). Meat quality, particularly flavor characteristics, significantly influences consumer purchasing decisions. Existing studies demonstrate that pork flavor is determined by multiple intrinsic factors, including breed, age, and pork cuts (3–6), as well as extrinsic factors such as rearing practices and cooking methods (7, 8). The presence and subsequent reactions of flavor precursor compounds govern the development of flavor. Primary precursors include lipid derivatives, free amino acids, reducing sugars, and thiamine, which undergo complex interactions during thermal processing or enzymatic degradation (9). Extensive research has demonstrated that lipid components, including fatty acids (FAs), triglycerides, and phospholipids, serve as essential precursors in the formation of flavor compounds through various oxidative and thermal degradation pathways (10). The fatty acid composition plays a pivotal role in the formation of volatile flavor compounds in meat products (11). Notably, both the degree of saturation and concentration of fatty acids substantially determine the resulting flavor characteristics. Comparative analyses reveal that the unsaturated fatty acids exhibit markedly higher susceptibility to oxidative degradation than their saturated counterparts, consequently generating a more diverse array of volatile flavor compounds during thermal processing and storage (12).

FAs are aliphatic hydrocarbon chains primarily composed of carbon (C), hydrogen (H), and oxygen (O), characterized by the presence of a carboxyl group. Based on their association with other functional groups (e.g., glycerol, phosphate, carbohydrates, or proteins), FAs are classified into two categories: free fatty acids (FFAs) and esterified fatty acids (EFAs). FFAs are also termed nones-terified fatty acids (NEFAs). NEFAs serve as fundamental structural units of glycerides, phospholipids, glycolipids, lipoproteins, and other biomolecules, playing critical roles in various biological processes. For instance, under enzymatic catalysis, EFAs can be hydrolyzed from these molecules to form FFAs, which participate in energy production through *β*-oxidation, signal transduction, and the synthesis of hormones (13, 14). In terms of meat quality, existing studies have demonstrated that compared to triglycerides and phospholipids, FFAs are more prone to oxidation during cooking and serve as precursors for aroma-active compounds that contribute to beef flavor (15, 16). Furthermore, recent research has revealed that specific FFAs (e.g., oleic acid, linoleic acid, and stearic acid) can modulate taste perception through interactions with taste receptors (CD36 and GPR120) located in taste buds (17). These findings highlight the critical role of FFAs in determining meat flavor profiles and overall quality. However, current research on fatty acids’ role in meat quality has predominantly examined total fatty acid profiles rather than specifically investigating FFAs (3, 5, 18–20). Unlike total fatty acids, FFAs represent the metabolically active pool that can directly participate in flavor formation without requiring prior enzymatic hydrolysis. This oversight significantly constrains our mechanistic understanding of flavor development during cooking and processing. Furthermore, critical knowledge gaps remain regarding the precise composition of FFAs in different pork cuts and their quantitative contribution to the generation of key flavor compounds. To address these gaps, this study targets FFAs to elucidate their cut-specific distribution patterns and establish direct associations with volatile flavor compounds, thereby complementing previous phospholipidomics and multi-omics investigations.

Therefore, the objective of this study was to systematically investigate the FFAs profiles and volatile metabolite compositions across four anatomically distinct pork cuts (*longissimus thoracis* (LT), *semimembranosus* (SMM), *psoas major* (PS), and *semitendinosus* (SMT)). Through advanced chromatographic and mass spectrometric analyses, we identified and quantified the key FFAs and characteristic volatile flavor compounds in each pork cut. Subsequent correlation analysis revealed significant FFA-flavor associations. These findings provide novel mechanistic insights into how muscle-specific FFA distributions may regulate flavor development through both oxidative degradation and potential receptor-mediated pathways.

## 2 Materials and methods

### 2.1 Materials

Shanxia long black pigs (SXLB) from the same commercial farm were selected, all raised under standardized feeding conditions with identical complete feed formulations throughout the fattening period (21). After reaching market weight, six genetically half-sibling sows with homogeneous carcass weights (mean ± SD: 93.40 ± 2.93 kg) and matched ages (226 ± 5 days) were selected for subsequent sampling. The selected pigs were fasted for 12 h prior to humane slaughter at a certified commercial abattoir following standard industry protocols. Immediately post-slaughter, four representative pork cuts were collected: *longissimus thoracis* (LT), *semimembranosus* (SMM), *psoas major* (PS), and *semitendinosus* (SMT). All samples were meticulously trimmed of epimysial fat and connective tissue, flash-frozen in liquid nitrogen within 15 min of collection, and subsequently stored at −80 °C until multi-omics analysis. This study was conducted in full compliance with international animal welfare standards and approved by the Institutional Animal Care and Use Committee (IACUC) of Jiangxi Agricultural University and Nanchang Normal University following the Guidelines for Ethical Review of Laboratory Animal Welfare (GB/T 35892-2018) issued by the Ministry of Agriculture and Rural Affairs of China. All experimental protocols were designed to minimize animal discomfort and were performed by trained personnel under veterinary supervision.

### 2.2 Free fatty acids analysis

The frozen sample (−80 °C) was cryogenically pulverized in liquid nitrogen (3 min) using a high-frequency grinder (30 Hz, 1.5 min), after which 50 mg aliquots were precisely weighed into EP tubes, homogenized with 700 μL methanol-based extraction solution containing the internal standard, and subjected to sequential vortexing (2,500 rpm, 10 min) and ultrasonic treatment (15 min) in a 4 °C water bath (22, 23). The sample extract was centrifuged (4 °C, 4200 rpm, 5 min), and 500 μL supernatant was transferred to a glass bottle containing 200 μL of 0.05 M methanolic NaOH. After nitrogen blow-down evaporation, the residue was reconstituted in 400 μL of 0.4 M methanolic NaOH and derivatized at 70 °C for 10 min. Following cooling to room temperature, reaction products were extracted using a liquid–liquid partition with 500 μL dichloromethane and 200 μL ddH₂O (vortexed at 2500 rpm for 5 min, then centrifuged at 4200 rpm, 4 °C for 5 min). The upper layer (300 μL) was transferred to a new glass bottle containing 300 μL of 2 M methanolic HCl for acid-catalyzed methylation at 70 °C for 20 min. After cooling, the methyl esters were extracted with 500 μL n-hexane and 300 μL ddH₂O (vortexed at 2500 rpm for 5 min, then centrifuged at 4200 rpm, 4 °C for 5 min). The hexane layer containing free fatty acid methyl ester was collected for GC–MS analysis (24).

Standard solutions of free fatty acids were prepared at concentrations of 0.004, 0.01, 0.02, 0.04, 0.1, 0.2, 0.4, 1, 2, 4, 10, 15, and 20 μg/mL. To each standard solution, isotope internal standards were added at fixed concentrations: 2 μg/mL deuterated octanoic acid (C8:0-d15), 2 μg/mL deuterated undecanoic acid (C11:0-d21), 2 μg/mL deuterated heptadecanoic acid (C17:0-d33), 5 μg/mL 13C-labeled linoleic acid (13C18:2n6c), and 5 μg/mL deuterated docosanoic acid (C22:0-d43). After derivatization with hydrochloric acid in methanol, the resulting derivatives were analyzed by GC–MS to acquire chromatographic peak intensities for each concentration. Calibration curves were constructed for each fatty acid by plotting the concentration ratio (standard/internal standard) on the *x*-axis against the peak area ratio (standard/internal standard) on the *y*-axis.

The sample derivatives were analyzed using an Agilent 7890B gas chromatography system coupled with an Agilent 7000D triple quadrupole mass spectrometer (Agilent Technologies, https://Agilent.com.cn/). Chromatographic separation was achieved on a DB-5MS capillary column (30 m × 0.25 mm × 0.25 μm, Agilent) with ultra-high purity helium (> 99.999%) as carrier gas at a constant flow rate of 1.0 mL/min. The oven temperature program was initiated at 50 °C (hold 1.5 min), then ramped as follows: 30 °C/min to 170 °C, 15 °C/min to 200 °C, 5 °C/min to 220 °C (hold 2 min), 8 °C/min to 228 °C, 20 °C/min to 240 °C (hold 2 min), 25 °C/min to 265 °C (hold 2 min), and finally 30 °C/min to 280 °C (hold 2 min). Samples (2 μL) were injected in split mode (5:1 ratio) with the injector maintained at 250 °C. Mass spectrometric detection was performed using electron ionization (EI) at 70 eV with the ion source temperature set to 230 °C. The transfer line and quadrupoles were maintained at 280 °C and 150 °C, respectively. Data acquisition in selected ion monitoring (SIM) mode commenced after a 4 min solvent delay.

The integrated peak areas of all detected samples were substituted into the linear equation of the standard curve for calculation, and further substituted into the calculation formula to finally obtain the content data of the target substance in the actual samples. Note: Unit conversions have already been incorporated into the calculation formula; directly substituting the corresponding values yields the sample content. The content of free fatty acids in the sample (μg/g) is calculated as follows:


$$\text{Content}(\frac{\mathrm{\mu g}}{g})=\frac{c\times v_{1}\times v_{3}}{v_{2}\times m\times 1000}$$

Where c is the concentration (μg/mL) obtained by substituting the integrated peak area of the sample into the standard curve; v_1_ is the volume of sample extraction solution (μL); v_2_ is the volume of collected supernatant (μL); v_3_ is the volume of reconstitution solution (μL); m is the sample mass (g).

### 2.3 Analysis of volatile flavour compounds

For volatile compound analysis, the preparation and extraction were completed by Wuhan Metware Biotechnology Co., Ltd. Briefly, the frozen samples were cryogenically homogenized using liquid nitrogen. Aliquots (200 mg) of the resulting powder were immediately transferred to 20 mL headspace vials (Agilent, Palo Alto, CA, USA) containing 200 mg sodium chloride to quench enzymatic activity, and the internal saturated solution was a p-Xylene-d10 standard solution (10 μg/mL). Vials were hermetically sealed with crimp-top caps fitted with TFE-silicone septa (Agilent). Prior to solid-phase microextraction (SPME), samples were equilibrated at 60 °C for 5 min, followed by headspace extraction using a 120 μm DVB/CAR/PDMS fiber (divinylbenzene, carbon wide range, polydimethylsiloxane, Agilent) exposed to the vial headspace for 15 min at 60 °C under continuous agitation. The details of the extraction methods refer to previous studies (25, 26).

Volatile organic compounds (VOCs) were thermally desorbed from the SPME fiber in the GC injection port at 250 °C for 5 min (splitless mode). Compound separation was performed using an Agilent 8,890 GC system coupled with a 7000D triple quadrupole mass spectrometer, employing a DB-5MS capillary column (30 m × 0.25 mm × 0.25 μm; 5% phenyl-polymethylsiloxane stationary phase). Ultra-high purity helium carrier gas was maintained at a constant linear velocity of 1.2 mL/min. The GC oven temperature program was initiated at 40 °C (hold 3.5 min), then ramped at 10 °C/min to 100 °C, 7 °C/min to 180 °C, and 25 °C/min to 280 °C (final hold 5 min). Mass spectrometric detection was conducted in electron ionization (EI) mode at 70 eV, with the ion source, quadrupole, and transfer line maintained at 230 °C, 150° C, and 280 °C, respectively. Data acquisition was performed in selected ion monitoring (SIM) mode for targeted compound identification and quantification.

The mass spectrometry raw data were processed using Agilent MassHunter Workstation software (version B.08.00) for both compound identification and semi-quantitative analysis. Volatile compounds were quantified by normalizing their chromatographic peak areas against an internal reference standard using the following equation:


$$X_{i}=\frac{V_{s}\times C_{s}}{M}\times \frac{I_{i}}{I_{s}}\times 10^{-3}$$

in which X_i_ is the content of target compound i in the sample (μg/g); *V_s_* is the volume of internal standard added (μL); C_s_ is the concentration of internal standard (μg/mL); M is the sample mass (g); I_i_ is the peak area of target compound i; I_s_ is the peak area of internal standard.

### 2.4 Relative odor activity value (rOAV) analysis

To elucidate the contribution of individual VOCs to the overall aroma profile, the relative odor activity value (rOAV) approach was applied to identify key aroma-active components. Based on the semi-quantification of various volatile substances in Section 2.3. The rOAV was calculated using the following equation (27):


$$\text{rOAVi}=\frac{C_{i}}{T_{i}}$$

In which rOAV_i_ is the relative aroma activity value of a given compound i; Ci is the concentration of compound i (μg/g); T_i_ is the water-based odor detection threshold of compound i (μg/g), which is obtained from the literature (28), published book (29) and also the National Standard of the People’s Republic of China GB/T 15549-2022/ISO 5496:2006 (typically 20–25 °C). In general, rOAV ≥ 1 indicates that the compound contributes directly to the sample flavor (30).

### 2.5 Data analysis

One-way analysis of variance (ANOVA) was used to determine the difference between means (*p* < 0.05). Multivariate statistical analyses were conducted using R (version 4.2.0) with the following workflow: First, unsupervised pattern recognition was performed through principal component analysis (PCA) and hierarchical cluster analysis (HCA) using the R packages. For supervised modeling, the data were preprocessed by log2 transformation followed by mean-centering prior to orthogonal projections to latent structures-discriminant analysis (OPLS-DA) using the *ropls* package. Model validity was verified through a 200-cycle permutation test to prevent overfitting. Significant differential metabolites between comparison groups were identified based on dual criteria: variable importance in projection (VIP) scores > 1.0 from the OPLS-DA model and |Log2Fold – change| ≥ 1.0 from univariate analysis. Despite the low *Q*^2^ values for the OPLS-DA models, differential metabolites were screened using the same criteria as an exploratory strategy.

## 3 Results

### 3.1 Free fatty acid composition in different cuts of pork

To accurately quantify free fatty acids in the samples, an isotope internal standard method was used in this study. After derivatization with hydrochloric acid in methanol, calibration curves were constructed by plotting the concentration ratio (standard/internal standard) on the *x*-axis against the peak area ratio (standard/internal standard) on the *y*-axis. Table 1 provides the resulting regression equations, limits of detection (LOD), and limits of quantification (LOQ). The quantitative distribution and relative proportions of individual FFA among the four different pork cuts, including the *longissimus thoracis* (LT), *semimembranosus* (SMM), *psoas major* (PS), and *semitendinosus* (SMT) muscle tissues were summarized in Table 2. A total of 35 FFAs were detected, and the FFAs’ composition in the four pork cuts was similar, while the relative content varied slightly. Although there were no significant inter-group differences in saturated fatty acid (SFA) composition (*p* > 0.05), the LT muscle showed a consistent but non-significant trend toward higher SFA percentages (mean ± SD: 27.33% ± 0.67%) relative to the other three muscle groups (range: 25.79% ± 2.61 to 26.01% ± 1.00%). Monounsaturated fatty acid (MUFA) levels in LT were significantly elevated compared to SMM (*p* < 0.05) but no significant difference versus the other two muscle groups (*p* > 0.05). Conversely, polyunsaturated fatty acid (PUFA) content in LT was significantly lower than that of the SMM (*p* < 0.05). PUFAs constituted the predominant fraction across all four pork cuts, accounting for > 40% of total free fatty acids, while SFAs and MUFAs showed comparable proportions at approximately 30% each.

**Table 1:** Calibration curves, LLOQ and ULOQ for free fatty acid analysis by GC–MS.

Index	Class	RT	Equation	*R* ^2^	Weighting	LLOQ	ULOQ
C6-0	Straight chain fatty acids	4.517266667	y = 865073.265632 x + 12035.419432	0.998601662	1/x	0.005	20
C8-0	Straight chain fatty acids	5.780616667	y = 1557483.138699 x + 7652.799440	0.993595398	1/x	0.005	20
C10-0	Straight chain fatty acids	6.9176	y = 0.426417 x − 4.246883E−004	0.997581525	1/x	0.01	20
C11-0	Straight chain fatty acids	7.500016667	y = 0.308614 x − 7.019860E−004	0.999195215	1/x	0.01	20
C12-0	Straight chain fatty acids	8.1261	y = 0.361314 x − 3.665835E−004	0.996886894	1/x	0.02	20
C13-1n1c	Monounsaturated fatty acids	8.789566667	y = 0.186424 x − 1.349179E−004	0.999214821	1/x	0.1	20
C13-0	Straight chain fatty acids	8.84265	y = 0.386676 x − 5.933957E−004	0.997544644	1/x	0.02	20
C14-1n5c	Monounsaturated fatty acids	9.5758	y = 0.148384 x − 3.667798E−004	0.995271698	1/x	0.1	20
C14-0	Straight chain fatty acids	9.668683333	y = 0.451073 x + 1.310899E−004	0.998190126	1/x	0.01	20
C15-1n5c	Monounsaturated fatty acids	10.52125	y = 0.082369 x − 1.053915E−004	0.997514302	1/x	0.1	20
C15-0	Straight chain fatty acids	10.62741667	y = 0.475245 x − 5.729210E−004	0.996060888	1/x	0.02	20
C16-1n7c	Monounsaturated fatty acids	11.49948333	y = 0.162551 x − 3.970639E−004	0.995273661	1/x	0.2	20
C16-0	Straight chain fatty acids	11.72218333	y = 0.513177 x + 0.031234	0.997928007	1/x	0.02	20
C17-0	Straight chain fatty acids	13.06368333	y = 0.489055 x − 9.409875E−004	0.998057571	1/x	0.02	20
C18-3n6c	Polyunsaturated fatty acids	13.91038333	y = 0.515474 x - 0.001259	0.999340819	1/x	0.5	20
C18-2n6c	Polyunsaturated fatty acids	14.16871667	y = 1.643548 x + 5.295035E−004	0.998941182	1/x	0.2	20
C18-1n9c	Monounsaturated fatty acids	14.2732	y = 0.699721 x − 5.718106E−005	0.998875188	1/x	0.1	20
C18-3n3c	Polyunsaturated fatty acids	14.2645	y = 0.751774 x − 7.455031E−004	0.998986959	1/x	0.2	20
C18-1n9t	Monounsaturated fatty acids	14.36608333	y = 0.748580 x − 0.001761	0.998302866	1/x	0.1	20
C18-0	Straight chain fatty acids	14.65923333	y = 0.510492 x + 0.013711	0.995376429	1/x	0.05	20
C19-0	Straight chain fatty acids	16.0486	y = 0.206110 x − 2.315233E−004	0.991235392	1/x	0.1	20
C20-4n6c	Polyunsaturated fatty acids	16.52393333	y = 0.213469 x − 4.400179E−004	0.998769619	1/x	1	20
C20-5n3c	Polyunsaturated fatty acids	16.61956667	y = 0.292281 x − 6.393992E−004	0.990363989	1/x	1	20
C20-3n6c	Polyunsaturated fatty acids	16.81645	y = 0.098703 x − 1.646573E−004	0.999455116	1/x	0.5	20
C20-2n6c	Polyunsaturated fatty acids	17.1399	y = 0.062142 x − 6.220811E−005	0.994337789	1/x	0.5	20
C20-1n9c	Monounsaturated fatty acids	17.2299	y = 0.217334 x − 2.052406E−004	0.996751937	1/x	0.1	20
C20-3n3c	Polyunsaturated fatty acids	17.24396667	y = 0.005301 x − 1.043879E−004	0.998158729	1/x	0.2	20
C20-1n9t	Monounsaturated fatty acids	17.32833333	y = 0.214926 x − 2.083558E−004	0.997548783	1/x	0.1	20
C20-0	Straight chain fatty acids	17.59835	y = 0.428620 x − 2.925816E−004	0.996040421	1/x	0.1	20
C22-6n3c	Polyunsaturated fatty acids	19.00366667	y = 0.157252 x + 2.679944E−004	0.998008736	1/x	1	20
C22-4n6c	Polyunsaturated fatty acids	19.0951	y = 0.210741 x − 6.923680E−004	0.996954998	1/x	1	20
C22-5n3c	Polyunsaturated fatty acids	19.17675	y = 0.359870 x − 0.001707	0.996238554	1/x	1	20
C22-1n9c	Monounsaturated fatty acids	19.6601	y = 0.240065 x + 0.001865	0.995577601	1/x	0.1	20
C22-0	Straight chain fatty acids	19.9769	y = 0.466818 x − 3.219793E−004	0.990984282	1/x	0.2	20
C24-1n9c	Monounsaturated fatty acids	21.95618333	y = 0.173204 x − 7.872340E−005	0.996435832	1/x	0.1	20

RT, Retention Time; Equation, Linear Equation; *R*^2^, Correlation coefficient; LLOQ, Lower limit of quantitation (ug/ML); ULOQ, Upper limit of quantitation (ug/ML).

**Table 2:** Comparative profile of free fatty acid composition across four pork cuts (expressed as percentage of total free fatty acids).

Free fatty acids	*Longissimus thoracis*	*Semimembranosus*	*Psoas major muscle*	*Semitendinosus*
Saturated fatty acid				
C6:0	ND	ND	0.02 ± 0.00a	0.01 ± 0.01a
C8:0	0.01 ± 0.00a	ND	0.01 ± 0.01a	0.01 ± 0.01a
**C10:0**	0.23 ± 0.04a	0.14 ± 0.07b	0.17 ± 0.04ab	0.23 ± 0.05a
C11:0	0.05 ± 0.02a	0.04 ± 0.02a	0.04 ± 0.01a	0.04 ± 0.01a
C12:0	0.16 ± 0.03a	0.15 ± 0.06a	0.14 ± 0.03a	0.17 ± 0.01a
C13:0	0.04 ± 0.01a	0.04 ± 0.02a	0.03 ± 0.01a	0.03 ± 0.01a
C14:0	1.14 ± 0.20a	0.92 ± 0.38a	0.99 ± 0.06a	1.01 ± 0.15a
C15:0	0.26 ± 0.09a	0.30 ± 0.24a	0.22 ± 0.03a	0.26 ± 0.08a
**C16:0**	18.41 ± 0.81a	16.63 ± 1.67b	17.50 ± 0.79ab	17.25 ± 0.89ab
C17:0	0.32 ± 0.07a	0.33 ± 0.08a	0.31 ± 0.05a	0.33 ± 0.08a
C18:0	6.47 ± 0.37a	7.04 ± 0.55a	6.36 ± 0.54a	6.44 ± 0.30a
C19:0	0.06 ± 0.01a	0.05 ± 0.01a	0.05 ± 0.01a	0.06 ± 0.02a
C20:0	0.11 ± 0.02a	0.10 ± 0.02a	0.11 ± 0.02a	0.11 ± 0.03a
C22:0	0.07 ± 0.02a	0.05 ± 0.02a	0.05 ± 0.01a	0.06 ± 0.01a
Monounsaturated fatty acid				
C13:1n1c	0.04 ± 0.01a	0.03 ± 0.01a	0.03 ± 0.01a	0.03 ± 0.01a
C14:1n5c	0.08 ± 0.02a	0.05 ± 0.02a	0.05 ± 0.01a	0.06 ± 0.01a
C15:1n5c	2.84 ± 0.82a	2.34 ± 0.82a	2.46 ± 0.88a	3.21 ± 0.40a
**C16:1n7c**	2.24 ± 0.45a	1.43 ± 0.56b	1.57 ± 0.18b	1.64 ± 0.23ab
**C18:1n9c**	20.98 ± 1.13a	16.08 ± 2.83b	19.03 ± 1.24ab	18.02 ± 1.86ab
C18:1n9t	2.97 ± 0.38a	2.57 ± 0.42a	2.46 ± 0.21a	2.57 ± 0.22a
**C20:1n9c**	0.43 ± 0.04ab	0.39 ± 0.05b	0.49 ± 0.06a	0.46 ± 0.07ab
C20:1n9t	0.09 ± 0.02a	0.07 ± 0.02a	0.08 ± 0.03a	0.08 ± 0.01a
C22:1n9c	0.59 ± 0.42a	0.20 ± 0.13a	0.40 ± 0.31a	0.41 ± 0.28a
**C24:1n9c**	0.05 ± 0.01a	0.03 ± 0.01b	0.04 ± 0.01ab	0.04 ± 0.01ab
Polyunsaturated fatty acid				
**C18:2n6c**	23.32 ± 2.06b	26.18 ± 2.79ab	28.34 ± 0.93a	28.54 ± 1.00a
**C18:3n3c**	0.84 ± 0.08b	0.82 ± 0.11b	1.07 ± 0.08a	1.05 ± 0.09a
C18:3n6c	0.21 ± 0.05a	0.18 ± 0.04a	0.19 ± 0.04a	0.19 ± 0.02a
C20:2n6c	0.51 ± 0.06b	0.63 ± 0.04a	0.62 ± 0.07a	0.63 ± 0.08a
C20:3n3c	1.33 ± 0.19a	1.16 ± 0.36a	1.22 ± 0.18a	1.53 ± 0.15a
**C20:3n6c**	1.80 ± 0.17b	2.16 ± 0.25a	1.59 ± 0.18b	1.58 ± 0.17b
**C20:4n6c**	12.25 ± 2.15b	17.34 ± 3.62a	12.03 ± 1.76b	11.62 ± 2.21b
C20:5n3c	0.89 ± 0.20a	1.00 ± 0.18a	0.80 ± 0.08a	0.78 ± 0.22a
C22:4n6c	0.50 ± 0.10a	0.49 ± 0.10a	0.57 ± 0.10a	0.57 ± 0.14a
C22:5n3c	0.66 ± 0.13a	0.64 ± 0.03a	0.63 ± 0.07a	0.69 ± 0.13a
C22:6n3c	0.94 ± 0.19a	0.80 ± 0.19a	0.77 ± 0.21a	0.77 ± 0.10a
SFA	27.33 ± 0.67a	25.79 ± 2.61a	25.97 ± 0.69a	26.01 ± 1.00a
**MUFA**	30.30 ± 1.82a	23.21 ± 4.23b	26.60 ± 2.35ab	26.54 ± 2.05ab
**PUFA**	42.37 ± 2.00b	51.01 ± 4.79a	47.43 ± 2.63ab	47.45 ± 2.85ab

Values represent mean ± standard deviation (*n* = 6) of six independent biological replicates. "ND" denotes concentrations below the lower limit of quantitation (LLOQ) (concentration in the extract below 5ng/ML). Significant differences (*p* < 0.05, one-way ANOVA with Tukey's *post-hoc* test) between pork cuts are indicated by distinct superscript letters (a–d) within each row. The null hypothesis of equivalent free fatty acids distribution between cuts was rejected at the 95% confidence level.

The bolded text indicates that there are significant differences in the free fatty acids among the four pork cuts. The same as below.

Palmitic acid (C16:0) was identified as the predominant SFA across all pork cuts, representing 16.63 − 18.41% of total FFAs. Statistical analysis revealed significantly lower palmitic acid content in SMM (16.63% ± 1.67%) compared to other muscle groups (17.25 − 18.41%, *p* < 0.05). In contrast, stearic acid (C18:0), another major naturally occurring SFA, showed a non-significant trend toward higher concentration in SMM (7.04% ± 0.55%) relative to other cuts (*p* > 0.05).

Oleic acid (C18:1n9c) represented the predominant MUFA across all pork cuts, accounting for 16.08 – 20.98% of total FFAs. Notably, LT, PS, and SMT demonstrated significantly higher oleic acid content compared to SMM (16.08% ± 2.83%) (*p* < 0.05). This elevated concentration in LT, PS, and SMT suggested its potential for enhanced flavor development, as linoleic acid could be involved as a potential contributor in the formation of aromatic volatile compounds during thermal processing.

Linoleic acid (C18:2n6c) emerged as the predominant PUFA in all pork cuts, accounting for > 23% of total FFAs. Notably, both PS and SMT muscles demonstrated significantly higher linoleic acid concentrations (28.34% ± 0.93 and 28.54% ± 1.00%, respectively) compared to LT (23.32% ± 2.06%; *p* < 0.05). This elevated PUFA profile likely enhances the flavor potential of PS and SMT through increased lipid oxidation-derived volatile compounds during cooking. In addition, the arachidonic acid (C20:4n6c) constituted 11.62 – 17.34% of total FFAs, making it the second most abundant PUFA across the four parts of pork. The relative content of C20:4n6c in the SMM was significantly higher than in the other parts (*p* < 0.05).

### 3.2 Analysis of volatile organic compounds among the four pork cuts

**Figure 1:**
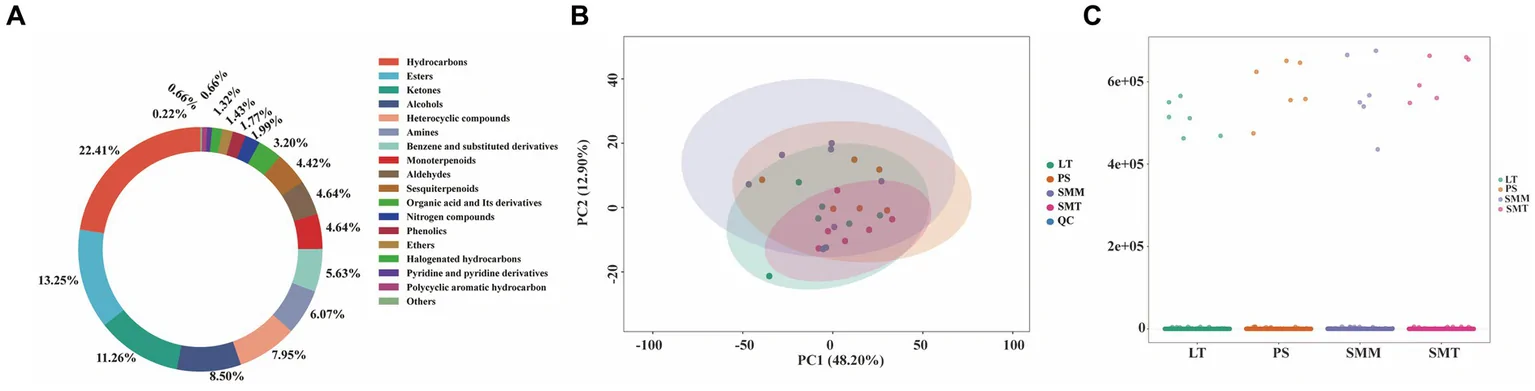
Summary of volatile organic compounds (VOCs) within the four pork cuts in SXLB pigs. **(A)** Distribution of VOCs from the four pork cuts. **(B)** PCA analysis of VOCs within the four pork cuts. PC1 and PC2 denote the first and second principal components, respectively, with the accompanying percentages reflecting the proportion of variance each component explains in the dataset. Each point in the plot represents an individual biological sample, and samples belonging to the same group are distinguished by identical colors **(C)** Scatter plot of relative odor activity values (rOAVs) for the four pork cuts. The horizontal axis represented different groups, whereas the vertical axis indicated the rOAVs of flavor compounds. LT, *Longissimus thoracis*; SMM, *Semimembranosus*; PS, *Psoas major muscle*; SMT, *Semitendinosus*. The same as below.

A total of 906 VOCs were identified in 24 samples (four groups with six biological replicates). Supplementary Table S1 showed all VOCs in the four cuts, while Supplementary Tables S2, S3 presented the relative content and rOAV of the detected VOCs, respectively. In terms of their chemical structures, these VOCs can be divided into 18 groups (Figure 1A). There were 203 hydrocarbons (22.41%), 120 esters (13.25%), 102 ketones (11.26%), 77 alcohols (8.50%), 72 heterocyclic compounds (7.95%), 55 amines (6.07%), 51 benzene and substituted derivatives (5.63%), 42 monoterpenoids (4.64%), 42 aldehydes (4.64%), 40 sesquiterpenoids (4.42%), 29 organic acid and its derivatives (3.20%), 18 nitrogen-containing compounds (1.99%), 16 phenolics (1.77%), 13 ethers (1.43%), 12 halogenated hydrocarbons (1.32%), 6 pyridine and pyridine derivatives (0.66%), 6 polycyclic aromatic hydrocarbon (0.66%) and 2 other compounds (0.22%). Chemical characterization of VOCs in pork revealed that hydrocarbons, esters, and ketones constituted the predominant components.

PCA enables rapid visualization of overall VOC differences between groups while simultaneously evaluating the degree of variation among samples within each group. The overlapping PCA score plot of the four muscle groups indicated relatively homogeneous VOC distributions among the four pork cuts (Figure 1B). Compared with the LT, the rOAV was obviously higher in SMM, PS, and SMT (Figure 1C).

### 3.3 Characterization and function analysis of differential VOCs

**Figure 2:**
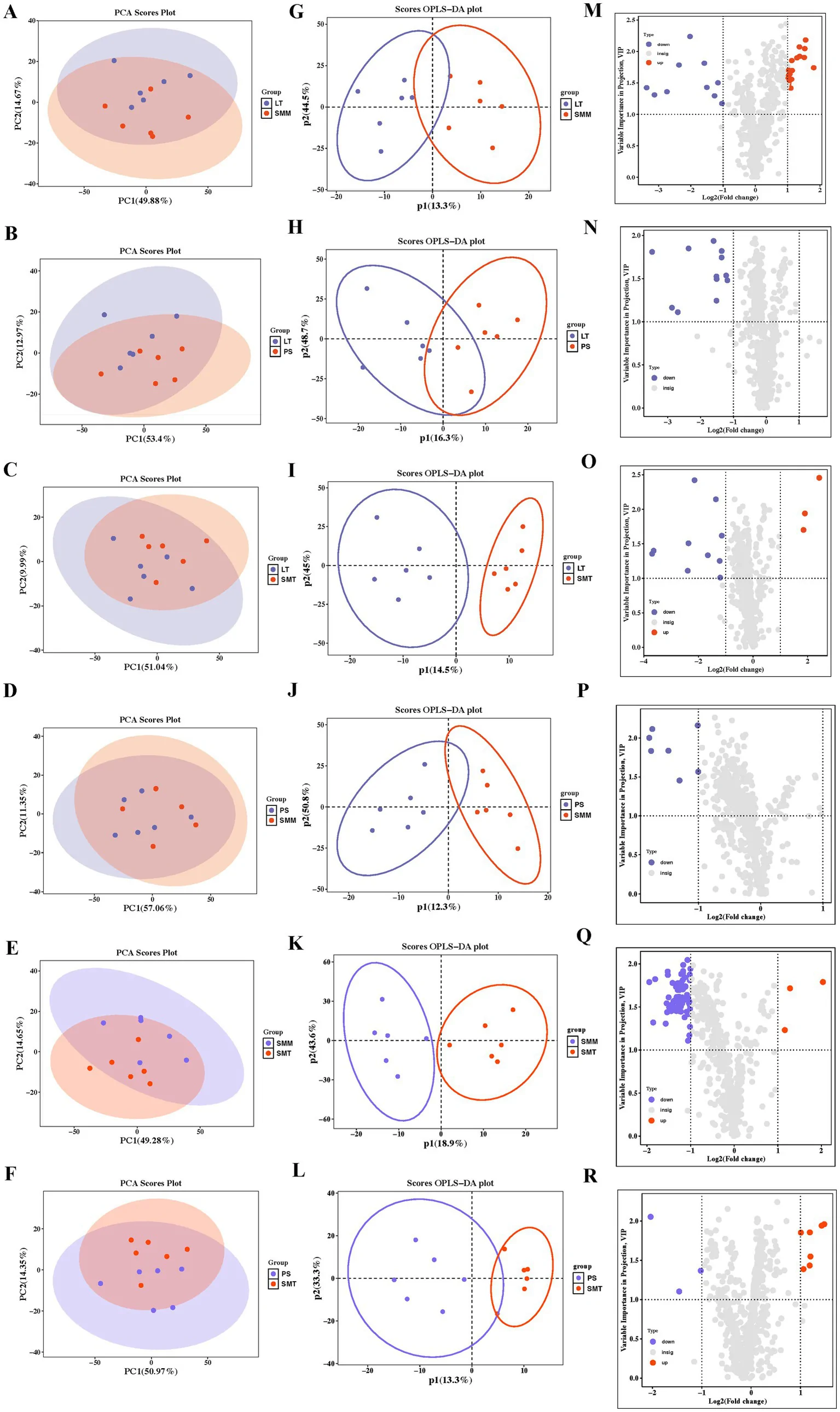
Identification of differential VOCs in the four port cuts. **(A–F)** PCA score of identified VOCs in LT vs. SMM, LT vs. PS, LT vs. SMT, SMM vs. PS, SMM vs. SMT, and PS vs. SMT, respectively. **(G–L)** OPLS-DA score of identified VOCs in LT vs. SMM, LT vs. PS, LT vs. SMT, SMM vs. PS, SMM vs. SMT, and PS vs. SMT, respectively. **(M–R)** Volcano plot showing VOCs in LT vs. SMM, LT vs. PS, LT vs. SMT, SMM vs. PS, SMM vs. SMT, and PS vs. SMT. VOCs that are significantly up-regulated or down-regulated are indicated in red and blue dots. VOCs without statistical significance are indicated by gray dots.

To characterize the differences in volatile organic compounds among the four pork cuts, a dual multivariate analytical approach incorporating PCA and OPLS-DA was employed for comparative modeling. The PCA results indicated considerable overlap among the sample distributions of LT, SMM, PS, and SMT, suggesting minimal differences in volatile metabolites between the pairwise comparison groups (Figures 2A–F). The OPLS-DA plots revealed that VOC differences among the compared groups were relatively pronounced, except for LT vs. PS and SMM vs. PS, which showed no significant difference (Figures 2G–L). The OPLS-DA models showed good performance with explained variation (R2Y) and prediction capability (Q2) values > 0.5 and R2Y > 0.8 for LT vs. SMM, LT vs. SMT, SMM vs. SMT, and PS vs. SMT (Supplementary Figure S1). These parameters confirmed good model fidelity for subsequent volatile flavor feature selection.

**Figure 3:**
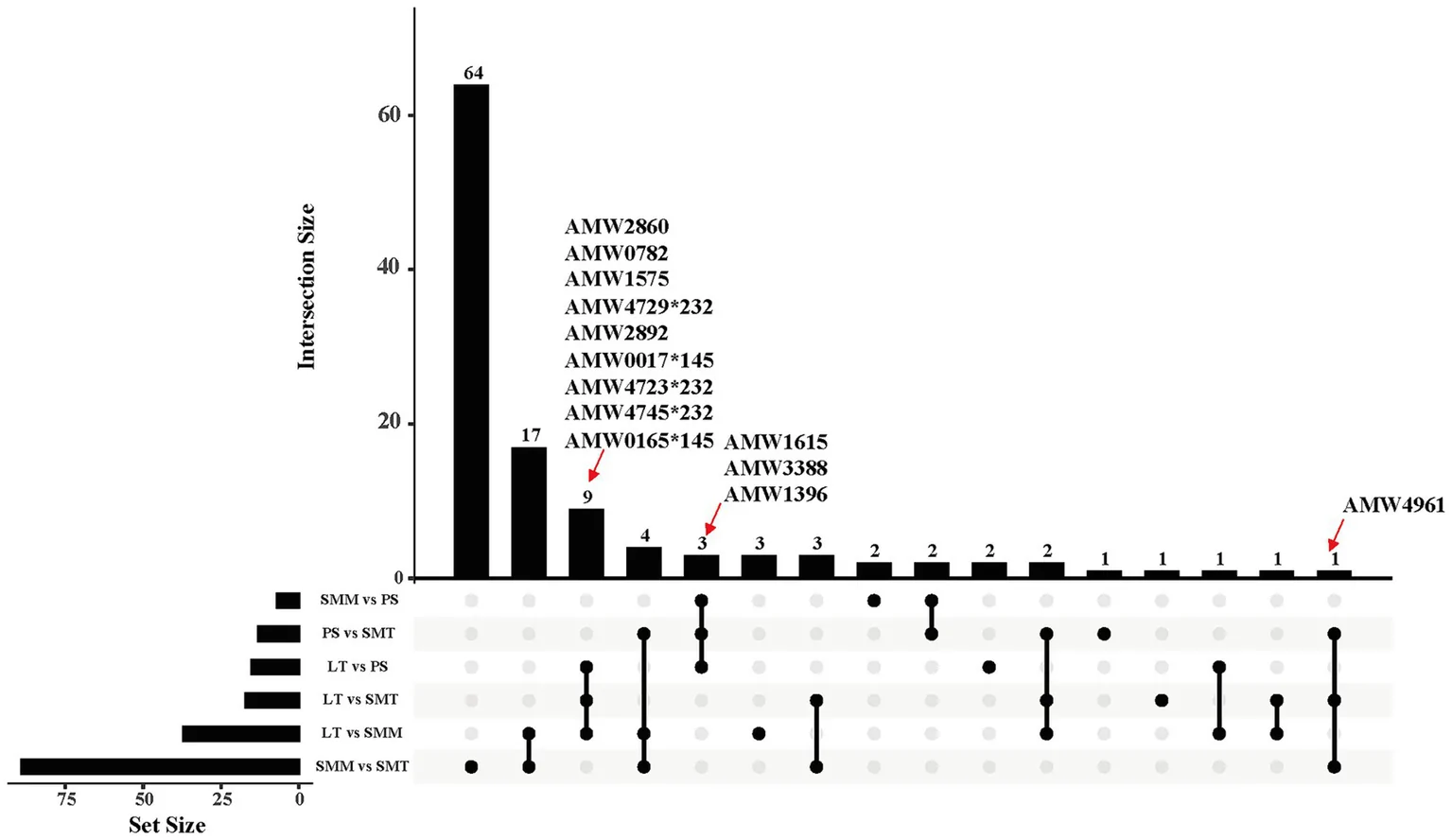
Upset Venn diagram analysis of the differential VOCs in the six compared groups.

Differentially accumulated VOCs among the four pork cuts were identified using the OPLS-DA model-derived VIP scores, with VIP > 1 and fold change thresholds ≥ 2 (up-regulated) or ≤ 0.5 (down-regulated) as the criteria. There were 22, 0, 4, 0, 5, and 10 up-regulated and 15, 15, 13, 7, 84, 3 down-regulated volatile compounds detected in “LT vs. SMM,” “LT vs. PS,” “LT vs. SMT,” “SMM vs. PS,” “SMM vs. SMT,” “PS vs. SMT” comparisons, respectively (Figures 2M–R; Supplementary Tables S4–S9). The quantitative analysis revealed that 77% of the total differential VOCs were down-regulated. The most significant VOC differences were observed between the SMM and SMT groups (*n* = 89), suggesting substantial flavor divergence between the two specific pork cuts. Then, the differentially accumulated VOCs were visualized by a heatmap within the comparisons (Supplementary Figures S2A–F), and the figures showed that the differential metabolites could separate most of the comparison groups well. The top VOCs ranked by VIP scores and log2-transformed fold changes (log2FC) were selected and visualized to highlight significant variations between groups (Supplementary Figures S2G–R). Further analysis of shared metabolites across all six comparison groups revealed distinct VOC profiles. As illustrated in Figure 3, the SMM vs. SMT comparison exhibited the highest number of differential VOCs (*n* = 64), suggesting significant flavor differentiation between these two pork cuts. Notably, we identified unique VOC signatures specific to individual cuts: LT contained 9 distinctive VOCs, including the succinimide, heptadecanoic acid-methyl ester, n-Hexadecanoic acid, (E)-2-Hexenoic acid, 4-Chlorobutyric acid, 4-isopropylphenyl ester, trans-2,9-anti-9,10-trans-1,10-Tricyclo[8.6.0.0 (2,9)]hexadeca-3,15-diene, (E)-3-Hexenoic acid, 2-Hexenoic acid, and cis-2,9-anti-9,10-cis-1,10-Tricyclo[8.6.0.0 (2,9)]hexadeca-3,15-diene, while PS and SMT had 3 (4-Octanone, propanal, propylhydrazone, H-Pyran-2-one) and 1 (Bicyclo[2.2.1]heptane-2,3-dione, 1,7,7-trimethyl-, (1S)) unique compounds, respectively.

**Figure 4:**
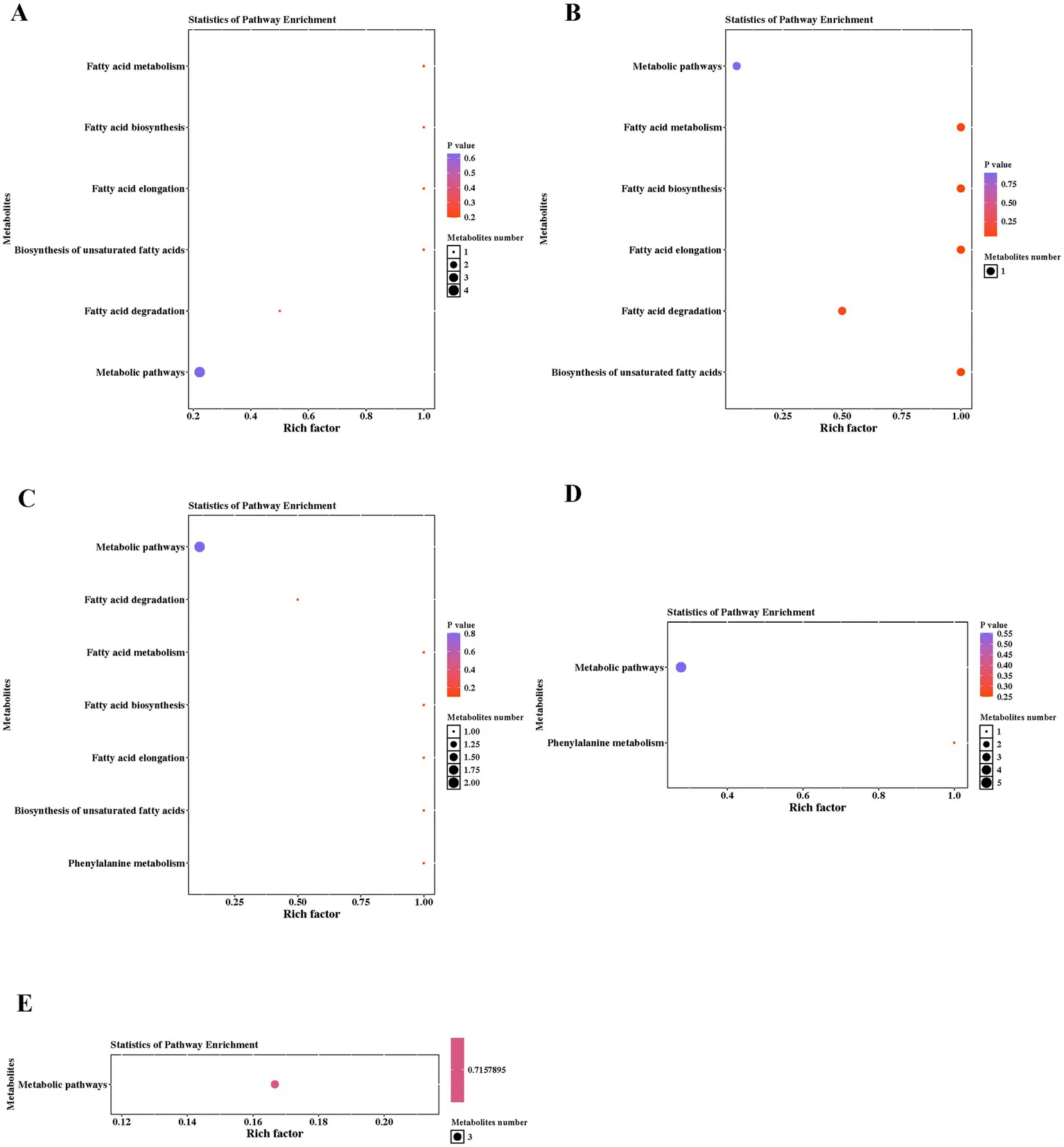
KEGG enrichment analyses of differential VOCs. **(A–E)** KEGG pathways in LT vs. SMM, LT vs. PS, LT vs. SMT, SMM vs. SMT, and PS vs. SMT, respectively. The *x*-axis represents the Rich Factor for each pathway, while the *y*-axis displays the pathway names ranked by *p*-value. The color of the dots corresponds to the *p*-value, with redder shades indicating greater enrichment significance. The size of the dots reflects the number of differential VOCs enriched in each pathway.

KEGG pathway analysis was employed to elucidate the biological functions and metabolic pathways associated with the differential VOCs. While no pathways showed significant enrichment (*p* > 0.05), several biologically relevant patterns emerged. The LT vs. SMM comparison showed enrichment in six fatty acid-related pathways (metabolism, biosynthesis, elongation, unsaturated fatty acid biosynthesis, degradation) and general metabolic pathways (all *p* > 0.05) (Figure 4A; Supplementary Table S10). A similar but less extensive pattern was observed in LT vs. PS, with all KEGG pathways remaining non-significant (*p* > 0.05) (Figure 4B; Supplementary Table S11). The LT vs. SMT comparison exhibited the broadest enrichment profile, encompassing all six pathways found in LT vs. SMM plus phenylalanine metabolism (all *p* > 0.05) (Figure 4C; Supplementary Table S12). More limited enrichment was detected in SMM vs. SMT (metabolic pathways and phenylalanine metabolism) and PS vs. SMT (metabolic pathways only), both with *p* > 0.05 (Figures 4D,E; Supplementary Tables S13, S14). No pathways were enriched in the SMM vs. PS comparison. These results collectively suggested a tendency toward differential regulation of fatty acid and amino acid metabolism across pork cuts, although the differences did not reach statistical significance. Integrated analysis of enriched differential VOCs across these metabolic pathways identified six key compounds: AMW1575 (n-hexadecanoic acid), AMW0301*058 (1,3-dimethyl-benzene), AMW0242*058 (p-Xylene), AMW2240*058 (o-Xylene), AMW0502 (Benzeneacetaldehyde), AMW1384 (4-hydroxy- benzenepropanoic acid).

### 3.4 Determination of key volatile flavor compounds among the four pork cuts

**Figure 5:**
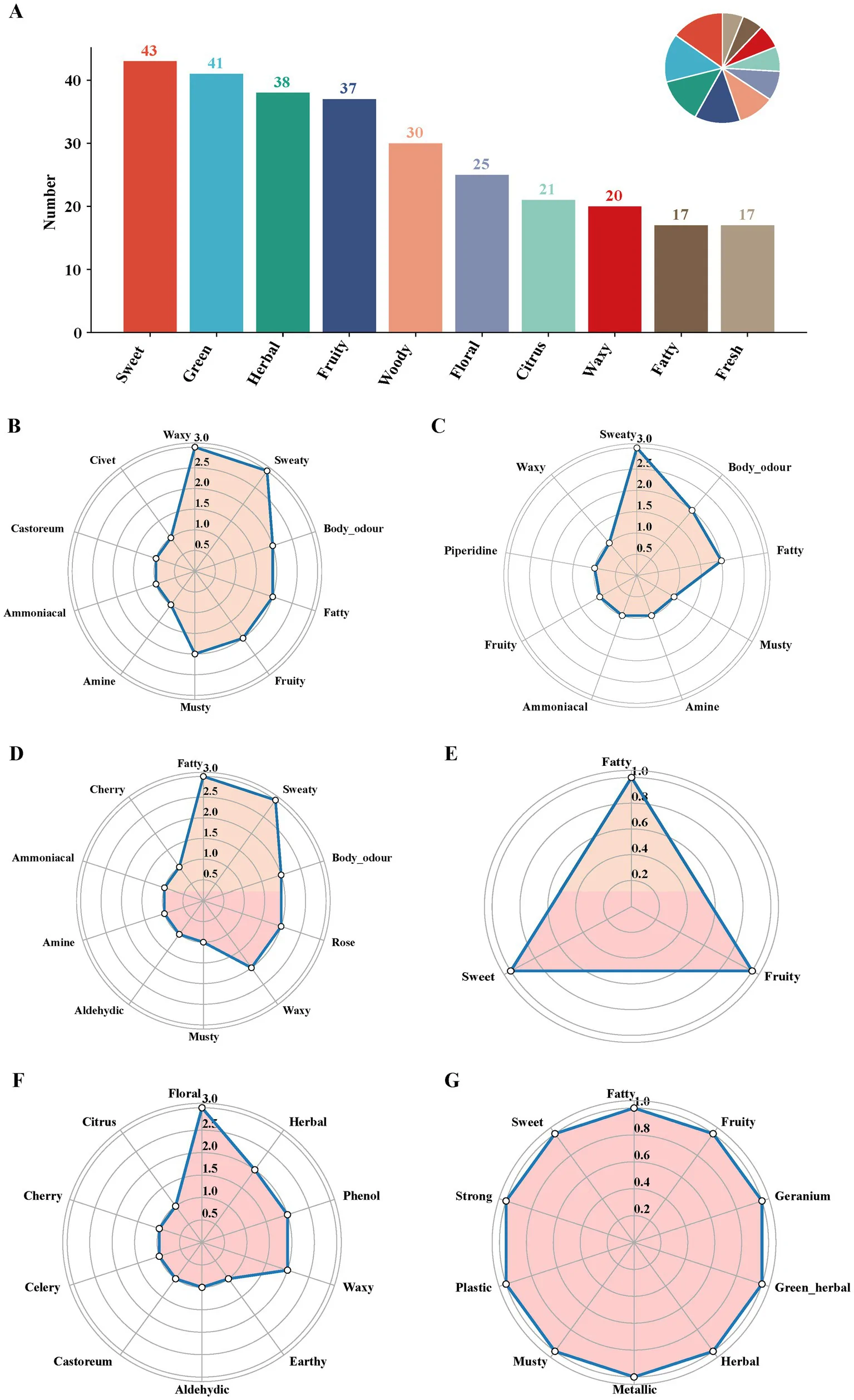
Identification of volatile flavor compounds (VFCs). **(A)** Statistical analysis of the top 10 flavor attributes in pork. The *X*-axis represents the flavor attributes, and the *Y*-axis indicates the number of corresponding metabolites. The pie chart in the upper-right corner illustrates the proportional distribution of the different flavor attributes. **(B–G)** Radar charts of sensory flavor characteristics for differential VFCs in LT vs. SMM, LT vs. PS, LT vs. SMT, SMM vs. PS, SMM vs. SMT, and PS vs. SMT. The outermost labels denote the sensory flavor attributes, and the numbers corresponding to the white points represent the counts of occurrences of each sensory flavor attribute.

**Figure 6:**
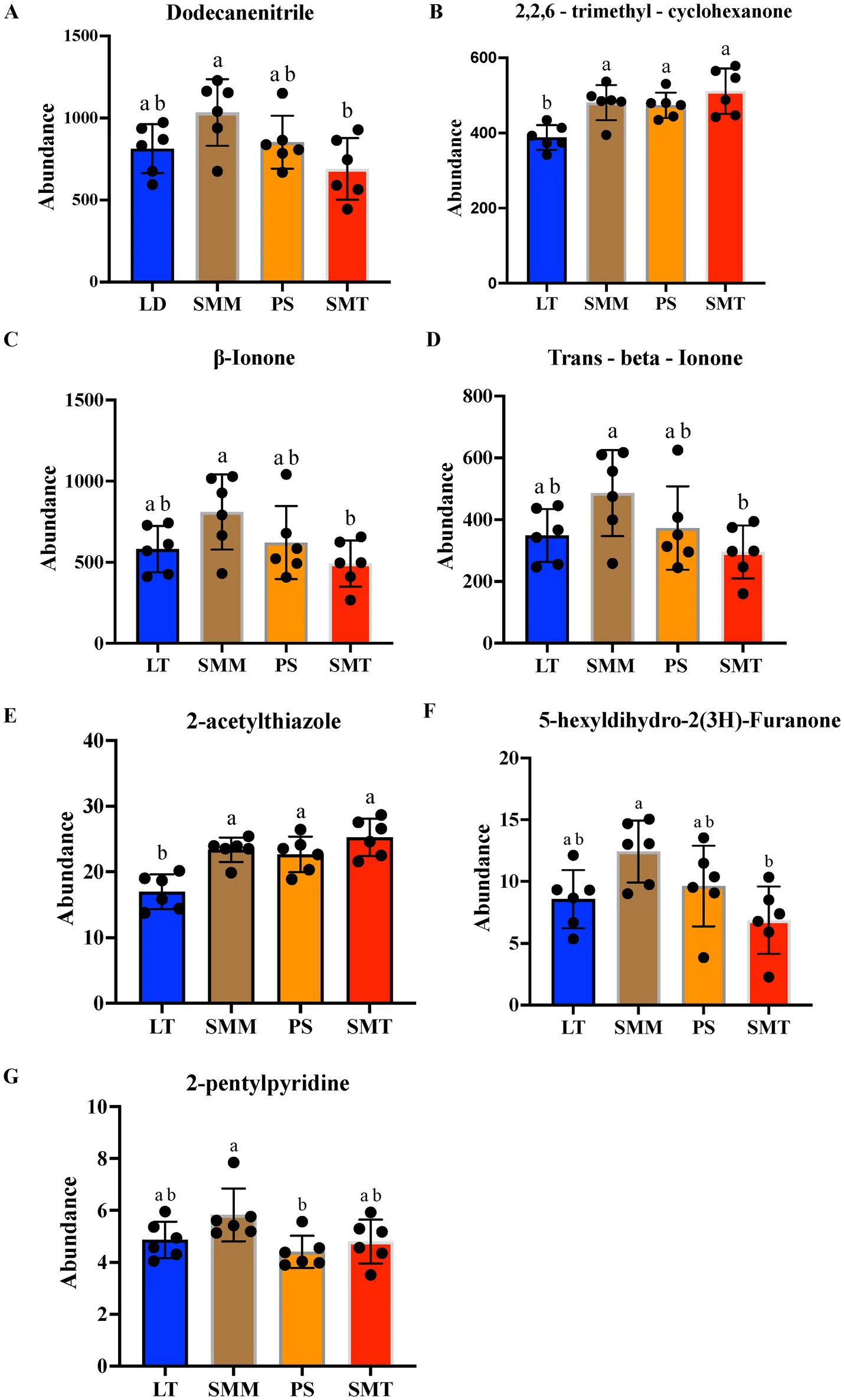
Comparison of the seven significantly differential flavor metabolites. **(A)** Dodecanenitrile. **(B)** 2,2,6-trimethyl-cyclohexanone. **(C)** *β*-Ionone. **(D)** trans-beta-Ionone. **(E)** 2-acetylthiazole. **(F)** 5-hexyldihydro-2(3H)-furanone. **(G)** 2-pentylpyridine. The *X*-axis represents the experimental groups, and the *Y*-axis indicates the relative abundance of the metabolites. Significant differences of metabolites abundance (*p* < 0.05, one-way ANOVA with Tukey’s post-hoc test) among pork cuts are indicated by distinct superscript letters (a–d).

A total of 200 volatile flavor compounds (VFCs) were identified and quantified with specific rOAV values, among which 150 were characterized with specific flavor attributes (Supplementary Table S15). Overall, the pork exhibited a complex flavor profile comprising sweet, green, herbal, fruity, woody, and floral notes (Figure 5A). Radar plots were generated to display the top 10 sensory flavor attributes (ranked by annotation frequency) derived from the screened differential VFCs in each comparison group (Figures 5B–G). These results demonstrated distinct VFC profiles across four pork cuts, indicating each possessed unique flavor characteristics. Given that all these differential VFCs exhibited rOAV < 1, they were unlikely to contribute to pork flavor profiles significantly. Therefore, we focused on VFCs with rOAV > 1, which were considered key flavor determinants. As shown in Table 3, a total of 31 VFCs demonstrated an rOAV > 1 in four pork cuts, indicating their potential as key odor contributors. While most VFCs did not exhibit significant abundance differences across the four pork cuts, seven metabolites were revealed with statistically significant variations in the pork cuts. These differentially VFCs include dodecanenitrile, 2,2,6-trimethyl-cyclohexanone, *β*-Ionone, trans-beta-Ionone, 2-acetylthiazole, 5-hexyldihydro-2(3H)-furanone, and 2-pentylpyridine (Figures 6A–G). Dodecanenitrile and 5-hexyldihydro-2(3H)-furanone showed significantly higher abundances in SMM compared to SMT (*p* < 0.05), but no significant differences versus PS and LT. Similarly, β-Ionone and trans-β-Ionone levels were significantly elevated in SMM over SMT (*p* < 0.05), while remaining comparable to PS and LT. In contrast, 2,2,6-trimethyl-cyclohexanone and 2-acetylthiazole exhibited their lowest abundances in LT (*p* < 0.05), whereas 2-pentylpyridine was significantly higher in SMM than in PS (*p* < 0.05), with no notable differences against LT and SMT. The results indicated distinct flavor profiles among the four pork cuts, with SMM showing the most abundant flavor characteristics. To explore the relationship between flavor compounds and FFAs, a correlation analysis was conducted based on 31 flavor compounds and 35 FFAs.

**Table 3:** Key volatile flavour compounds in different pork cuts and their corresponding rOAV values.

Index^a^	Compounds	LT	SMM	PS	SMT	Odor description^b^
AMW1123	Dihydro-2-methyl-3(2H)-furanone	512174.07	572212.32	584894.94	612836.05	Sweet, solvent, bread, buttery, nutty
AMW1942	5-ethyl-3-hydroxy-4-methyl-2(5H)-furanone	4212.72	4619.34	4405.92	4602.73	Sweet, fruity, caramel, maple
AMW0507*387	4-methoxy benzaldehyde	1066.76	1094.94	1043.33	1081.11	Sweet, powdery, mimosa, floral
AMW0978	**Dodecanenitrile**	**813.83**	**1034.08**	**852.74**	**690.01**	**Citrus, orange, peel, metallic, spicy**
AMW0590*101	2-ethyl-3,5-dimethyl-pyrazine	592.87	593.18	599.74	612.22	Burnt, almond, roasted, nutty
AMW0970	**2,2,6-trimethyl-cyclohexanone**	**388.05**	**480.62**	**473.73**	**511.26**	**Pungent, thujone, labdanum, honey**
AMW0647*142	**β-Ionone**	**581.5**	**810.15**	**621.53**	**492.20**	**Floral, woody, sweet, fruity, berry**
AMW2135*142	**Trans-beta-Ionone**	**348.9**	**486.09**	**372.92**	**295.32**	**Dry, powdery, floral, woody, orris**
AMW1099*069	(E,E)- 3,5-Octadien-2-one	164.02	169.47	161.96	160.64	Fruity, green, grassy
AMW2312	**2-Acetylthiazole**	**16.97**	**23.37**	**22.65**	**25.24**	**Nutty, popcorn, roasted, peanut**
AMW1298*051	3-Furanmethanol	17.76	17.90	17.54	17.70	–
AMW0940*166	D-carvone	11.97	12.25	11.65	12.04	Spice, minty, bread, caraway
AMW1064	3-hydroxy-4,5-dimethyl-2(5H)-furanone	9.99	9.75	8.91	10.03	Extremely sweet, strong caramel
AMW4855*262	2,3-diethyl-5-methyl-pyrazine	8.89	7.52	10.50	9.77	Earthy-like
AMW0911	cis-7-Decen-1-al	9.01	9.22	8.84	9.05	Citrus, aldehydic, cucumber
AMW1951	**5-hexyldihydro-2(3H)-furanone**	**8.58**	**12.43**	**9.64**	**6.87**	**Fresh, oily, waxy, peach, coconut**
AMW1092	1-(4,5-dihydro-2-thiazolyl)- ethanone	6.31	6.53	6.20	6.30	Corn, chip, taco, potato, toasted
AMW1195	Furaneol	4.63	5.40	5.40	5.73	Sweet, cotton, candy, caramel
AMW2151	Tetrahydro-6-methyl-2H-pyran-2-one	5.25	5.48	5.21	5.31	Creamy, fruity, coconut
AMW4899	**2-pentyl-pyridine**	**4.86**	**5.83**	**4.40**	**4.81**	**Fatty, tallow, green, pepper**
AMW2488*252	2-Methoxy-5-methylphenol	4.13	4.04	4.06	3.89	–
AMW1343	Eucalyptol	2.87	3.15	3.11	3.13	Eucalyptus, herbal, camphor
AMW0412	Decanal	3.25	3.65	3.17	2.96	Sweet, aldehydic, waxy, orange peel
AMW0565*101	3-ethyl-2,5-dimethyl-pyrazine	2.76	2.76	2.79	2.85	Potato, cocoa, roasted, nutty
AMW3385*351	Pentanoic acid, pentyl ester	2.54	2.94	2.2	2.07	Ripe fruit, apple
AMW2282*166	Carvone	1.79	1.83	1.74	1.8	Minty, licorice
AMW4919*166	(-)-Carvone	1.41	1.44	1.37	1.42	Sweet, spearmint, herbal, minty
AMW0523*389	(E)-2-Butenal	1.21	1.32	1.4	1.32	–
AMW0532*062	1-Nonene	1.33	1.65	1.74	1.30	–
AMW0233*125	Geraniol	1.25	1.28	1.19	1.24	Sweet, floral, fruity, rose, waxy
AMW0200	5-butyldihydro-2(3H)-furanone	1.17	1.32	1.20	1.11	Sweet, coconut, waxy, creamy

a The ID number of the specific metabolite. The bolded text indicates that there are significant differences in the metabolites among the four pork cuts (seen in Figure 7).

b The odor descriptions are from https://www.thegoodscentscompany.com/, https://perflavory.com/, and https://www.odour.org.uk/odour/index.html?i=1. The “–” means there is no detailed description of flavor attributes.

### 3.5 Correlation analysis of free fatty acids and key flavor compounds

Correlation analysis can be employed to examine the interrelationships between variables and identify flavor precursors derived from FFAs that influence pork flavor characteristics (31). Figure 7 showed the Pearson correlation analysis results of important FFAs and key flavor compounds. Statistical analysis revealed significant negative correlations between C8:0/C10:0 and several key compounds (*p* < 0.01), including AMW0978, AMW0647*142, AMW2135*142, and AMW1951, while other SFAs showed only weak correlations with the 31 identified key flavor compounds. Among MUFAs, C15:1n5c demonstrated significant positive correlations with four metabolites and a negative correlation with one (*p* < 0.01). Notably, C20:1n9c exhibited substantial correlations with 16 metabolites, predominantly negative, whereas C18:1n9c and C8:1n9t showed significant negative correlations with 5 - 6 metabolites each. PUFAs generally displayed significant to weak correlations with these metabolites. Specifically, C18:3n3c showed significant negative correlations with six metabolites, while C20:2n6C, C20:3n6, and C20:5n3C each demonstrated substantial positive correlations with more than five metabolites. These findings support an association between FFAs and the formation of specific aroma characteristics, although further experiments are required to establish causality.

**Figure 7:**
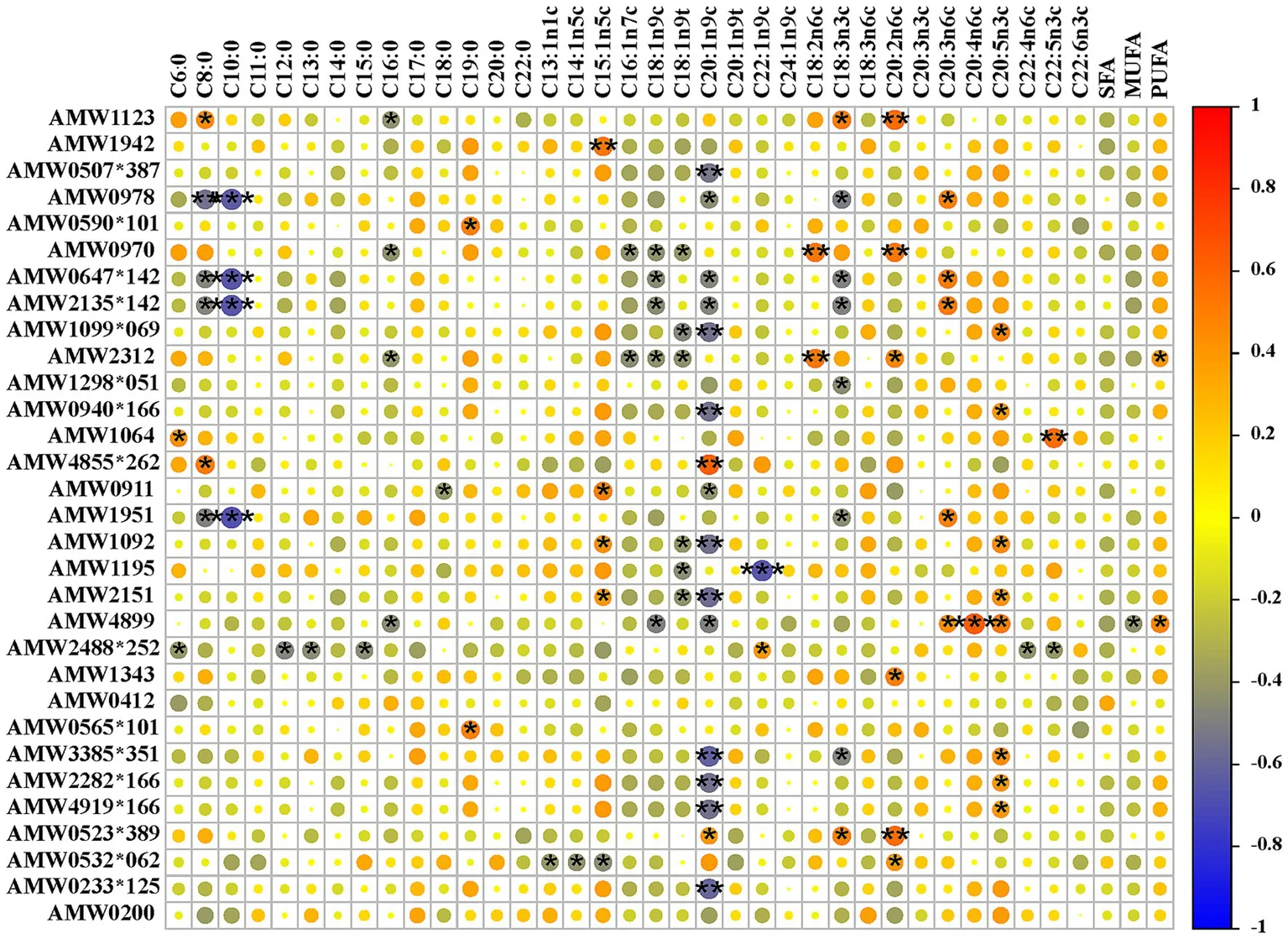
Pearson correlation between 31 identified VFCs (rOAV > 1) and 35 FFAs. *p < 0.05, **p < 0.01, ***p < 0.001.

## 4 Discussion

The flavor profile serves as a critical indicator for consumers when evaluating the quality of meat products (32, 33). Lipids, functioning as primary precursors of aroma compounds, significantly influence flavor formation, and the distinct aroma characteristics among different meat types are predominantly attributed to variations in lipid oxidation products. Extensive research has well-documented the significant contributions of fatty acids to meat quality traits, including meat color, pH, tenderness, flavor, etc. (5, 34). However, studies focusing specifically on the role of FFAs in the meat quality of different pork cuts remain relatively limited. Wei et al. identified 38 distinct FFAs in dry-cured hams derived from Dahe black pigs and their crossbred counterparts, demonstrating that oxidative degradation of key PUFAs - including trans-9-octadecenoic acid (C18:1n9t), cis-9,12-linoleic acid (C18:2n6c), *γ*-linolenic acid (C18:3n6), *α*-linolenic acid (C18:3n3), and arachidonic acid (C20:4n6) - played a pivotal role in generating the characteristic volatile profile of dry-cured hams (35). Current evidence indicates that compared with their esterified counterparts (e.g., triglycerides and phospholipids), FFAs exhibit higher susceptibility to oxidative degradation, thereby generating distinct flavor profiles in meat products (36). A total of 35 FFAs were detected in this study. Among them, palmitic acid (C16:0), oleic acid (C18:1n9c), linoleic acid (C18:2n6c), and arachidonic acid (C20:4n6c) each accounted for more than 10% of the total FFAs, and their levels differed significantly across the four pork cuts (*p* < 0.05). This proportional pattern of major FFAs was consistent with the findings of total FAs reported by Duan et al. (3). Notably, the LT muscle exhibited a relatively higher proportion of SFAs and MUFAs, along with a significantly lower proportion of PUFAs (*p* < 0.05). This distinct fatty acid profile may be attributed to its unique metabolic properties. Jeong et al. reported that in porcine LT muscle, greater density, relative area, and cross-sectional area of type I fibers were associated with higher intramuscular fat content, as well as elevated levels of SFAs and MUFAs. Conversely, larger cross-sectional area and relative area of type IIB fibers were correlated with reduced IMF content and an increased proportion of PUFAs (37). As is well recognized, oxidative muscles such as the PS and SMM generally exhibit a higher proportion of PUFAs compared to glycolytic muscles. This can be attributed to their elevated phospholipid content, which is intrinsically associated with greater mitochondrial density and increased membrane surface area within these more metabolically active tissues.

A total of 31 VFCs in this study exhibited a rOAV ≥ 1 in four pork cuts. Among these, seven metabolites showed significant differences across the four pork cuts, suggesting that they may be responsible for the major variations in odor observed. The presence of dodecanenitrile is more commonly recognized as an indicator of lipid oxidation, reflecting the progression of lipid degradation and associated flavor alterations, rather than as a direct contributor to the desirable aroma of meat products (38, 39). Moreover, dodecanenitrile showed consistent negative correlations with amino acid flavor precursors, suggesting its potential involvement in off-flavor formation (40). 2,2,6-trimethyl-cyclohexanone, a volatile ketone produced during lipid oxidation in meat, contributes only marginally to the overall aroma profile but is often employed as a chemical marker to evaluate the extent of lipid oxidation. *β*-Ionone (including its trans isomer) is a widely used aroma compound in the food and cosmetic industries and also serves as a key intermediate in the synthesis of vitamins A, E, and K (41). Although β-ionone is typically associated with fruity (e.g., apple, pear) and floral (e.g., violet) notes and contributes to the flavor of tea and dairy products (42, 43), it is rarely reported in the context of meat flavor. However, in cooked beef, β-ionone has been found to have relatively high flavor dilution factors compared with other compounds in each study, indicating that it is an active aroma compound in this matrix (44). Further experimental studies are needed to confirm its relevance and prevalence in meat products. *β*-Ionone and its isomer trans-β-ionone are primarily derived from the oxidative cleavage of carotenoids, especially β-carotene, via carotenoid cleavage dioxygenases or non-enzymatic autoxidation. In meat, their levels depend on carotenoid deposition in the muscle tissue, which can vary significantly between different muscle types due to differences in fiber composition, fat content, and metabolic activity (45, 46). SMM and SMT differ in their physiological function and biochemical environment, which may affect carotenoid accumulation and subsequent degradation to ionones. 2-acetylthiazole is characterized by a nutty and cereal-like flavor, imparting a distinct popcorn-like aroma. It has a low odor threshold (0.004–0.0041 mg/m^3^) and high odor intensity (47), and has been identified as a key aroma-active compound in various foods such as cooked red beans, ground beef, and pork liver (48). Research has shown that specific cooking methods, particularly stewing or boiling, promote the formation and detection of fat-derived lactones such as *γ*-hexalactone, due to prolonged interaction between lipids and juices. These compounds are known to contribute to the characteristic "rich" and "fatty" aroma profile of stewed meat (49). 2-pentyl-pyridine has been identified as a key shared volatile compound significantly contributing to the aroma quality of both chicken and pork (50). Buttery et al. identified several key volatiles responsible for the characteristic odor of mutton, among which 2-pentylpyridine was proposed as a candidate odor-active compound, likely formed through the reaction between ammonia and 2,4-decadienal, an oxidation product of lipids (51). Interestingly, these differential compounds exhibited the highest rOAV values in SMM, further confirming that SMM contains relatively more abundant flavor compounds, which is consistent with its widespread use in ham production.

The correlation analysis results between 35 FFAs and 31 key flavor metabolites showed that FFAs contributed to the meat flavor to varying degrees, especially C20:1n9c, which was significantly correlated with more than half of the key flavor metabolites. C20:1n9c (gondoic acid), a monounsaturated FFA, may indirectly contribute to meat flavor formation, potentially by serving as a precursor for volatile aroma compounds generated during thermal processing. Unlike short-chain FFAs that directly contribute to cheesy or rancid notes, gondoic acid undergoes lipid oxidation and degradation due to its unsaturated nature. This process, particularly through pathways such as *β*-oxidation and thermal cleavage, generates medium-chain aldehydes (e.g., nonanal, decanal), alcohols, and ketones that are key contributors to the desirable fatty, waxy, and sweet aroma notes in cooked meat. The extent of its contribution is highly dependent on the concentration of free gondoic acid released from phospholipids or triglycerides by lipase activity pre- and post-mortem. Furthermore, its oxidation products can interact with Maillard reaction intermediates, leading to the formation of additional heterocyclic compounds that enhance flavor complexity (52). The specific flavor profile influenced by gondoic acid is also modulated by factors such as animal diet, species, and cooking methods, as these determine its initial levels and the subsequent oxidative environment. Currently, there is limited direct research on the contribution of C20:1n9c to meat quality and flavor. Further investigation with larger sample sizes and more comprehensive data is warranted.

Based on this finding, we not only identified the common aroma-contributing substances and their important regulating FFAs across different pork cuts, but also revealed distinct differences in flavor compounds and FFAs among them. However, our experiments were somewhat one-sided because we used only one species with six individuals. Several limitations of this study should be acknowledged when interpreting the results. First, only one breed (Shanxia long black pig) was used, with six half-sibling females, limiting generalizability to other breeds, males, or the broader population. Second, pigs were raised under standardized feeding conditions; it is unknown whether the observed cut-specific FFA and VOC profiles persist under different management practices (e.g., free-range, organic feeding). Third, all animals were slaughtered at a fixed age (~230 days), so the effect of slaughter maturity on flavor precursors was not examined. Fourth, meat samples were analyzed after a defined cold storage period (e.g., 45 min at 4 °C); different aging times might alter FFA release and VOC generation. Therefore, the current conclusions are primarily applicable to the specific experimental conditions used in this study. Future research should include multiple breeds, both sexes, varied rearing systems, different slaughter ages, and multiple cold-storage durations to test the robustness and external validity of our findings. Such studies will help establish whether the cut-specific FFA–VOC correlations observed here represent a general principle of pork flavor generation or are context-dependent. Moreover, the flavor recombination and aroma dilution assays will be utilized to assess the contribution of key aroma compounds in meat quantitatively, and integrated multi-omics strategies and GC-O (gas chromatography-olfactometry) validation will be implemented to clarify the biosynthetic pathways of common volatile flavor substances. Anyway, our findings may help advance the understanding of the mechanisms involved in meat aroma quality and guide breeding and nutritional regulation strategies for high-quality meat.

## 5 Conclusion

Herein, FFAs and volatile profiles of four pork cuts from genetically half-sibling SXLB sows were analyzed using GC–MS. The results revealed significant differences in the levels of ten FFAs, including MUFA and PUFA, among the four pork cuts. Notably, the LT exhibited significantly higher contents of both SFA and MUFA compared to the SMM, PS and SMT (*p* < 0.05). VOC profiling identified 9, 3, and 1 unique volatiles in LT, PS, and SMT, respectively. Significantly differentiated VFCc across the four cuts included dodecanenitrile, 2,2,6-trimethylcyclohexanone, *β*-ionone, trans-beta-ionone, 2-acetylthiazole, 5-hexyldihydro-2(3H)-furanone, and 2-pentylpyridine, which may collectively contribute to the distinct flavor profile of SMM. Subsequent correlation analysis indicated that FFAs contribute variably to flavor formation, with C20:1n9c playing a particularly important role in shaping the volatile characteristics of pork. Together, these findings suggest a potential association between flavor-related compounds, FFAs, and different pork muscles. However, as this study primarily focused on raw meat composition, raw meat metabolites may only serve as indicators of potential flavor precursors rather than direct determinants of final aroma. Further investigation is therefore needed, including the analysis of additional sensory and chemical indicators, as well as the impact of different cooking processes on the final flavor profile. In particular, studies involving controlled thermal processing are required to establish direct links between precursor levels and cooked meat aroma.

## Data Availability

The original contributions presented in the study are included in the article/Supplementary material, further inquiries can be directed to the corresponding authors.

## Supplementary material

Supplementary Table S1 Quantitative analysis of the 906 volatile organic compounds.

Supplementary Table S2 Relative content of the 906 volatile organic compounds.

Supplementary Table S3 Relative odor activity value (rOAV) of the 906 volatile organic compounds.

Supplementary Table S4 Identification of differentially accumulated VOCs in LT vs SMM.

Supplementary Table S5 Identification of differentially accumulated VOCs in LT vs PS.

Supplementary Table S6 Identification of differentially accumulated VOCs in LT vs SMT.

Supplementary Table S7 Identification of differentially accumulated VOCs in SMM vs PS.

Supplementary Table S8 Identification of differentially accumulated VOCs in SMM vs SMT.

Supplementary Table S9 Identification of differentially accumulated VOCs in PS vs SMT.

Supplementary Table S10 KEGG analysis of differentially accumulated VOCs in LT vs SMM.

Supplementary Table S11 KEGG analysis of differentially accumulated VOCs in LT vs PS.

Supplementary Table S12 KEGG analysis of differentially accumulated VOCs in LT vs SMT.

Supplementary Table S13 KEGG analysis of differentially accumulated VOCs in SMM vs SMT.

Supplementary Table S14 KEGG analysis of differentially accumulated VOCs in PS vs SMT.

Supplementary Table S15 Details of the 200 volatile flavor compounds.

SUPPLEMENTARY FIGURE S1 **(A–F)** Details about the OPLS-DA model of the volatile organic compounds in LT vs SMM, LT vs PS, LT vs SMT, SMM vs PS, SMM vs SMT, and PS vs SMT, respectively.

SUPPLEMENTARY FIGURE S2 **(A–F)** Heatmap of differential VOCs in LT vs SMM, LT vs PS, LT vs SMT, SMM vs PS, SMM vs SMT, and PS vs SMT, respectively. **(G–L)** The top VOCs ranked by VIP scores in LT vs SMM, LT vs PS, LT vs SMT, SMM vs PS, SMM vs SMT, and PS vs SMT, respectively. **(M–R)** The top VOCs ranked by log2-transformed fold changes (log2FC) LT vs SMM, LT vs PS, LT vs SMT, SMM vs PS, SMM vs SMT, and PS vs SMT, respectively.
