# Supplementary material for: Correlations between free fatty acids and volatile compounds in four pork cuts from Shanxia long black pigs: implications for cut-specific flavor generation
Source: Front Nutr. 2026 Aug 13;13:1840577. doi: 10.3389/fnut.2026.1840577 (PMC13518193; doi:10.3389/fnut.2026.1840577)
Supplement: Supplementary file 1 [file Supplementary_file_1.docx]

Supplementary figures

Figure S1

Figure S2
